# Integration of a Patient-Centered mHealth Intervention (Support-Moms) Into Routine Antenatal Care to Improve Maternal Health Among Pregnant Women in Southwestern Uganda: Protocol for a Randomized Controlled Trial

**DOI:** 10.2196/67049

**Published:** 2025-03-19

**Authors:** Esther Cathyln Atukunda, Godfrey Rwambuka Mugyenyi, Jessica E Haberer, Mark J Siedner, Angella Musiimenta, Josephine N Najjuma, Celestino Obua, Lynn T Matthews

**Affiliations:** 1 Mbarara University of Science and Technology Mbarara Uganda; 2 Department of Medicine and Center for Global Health Harvard Medical School Massachusetts General Hospital Boston, MA United States; 3 Division of Infectious Diseases School of Medicine University of Alabama at Birmingham Birmingham, AL United States

**Keywords:** social support, intervention development, maternal health, antenatal care attendance, skilled births, Uganda

## Abstract

**Background:**

Mobile health (mHealth) interventions that leverage social support (SS) can improve partner involvement and pregnancy experiences and promote antenatal care (ANC) attendance and skilled births. In our previous studies, we used behavioral frameworks to develop a user-centered mHealth-based, audio SMS text messaging app to support pregnant individuals to use maternity care services in rural Uganda (Support-Moms app). In our pilot study, we observed high intervention uptake, acceptability, and feasibility, as well as increased ANC attendance and skilled births.

**Objective:**

With the promising pilot data, we propose a type 1 hybrid implementation-effectiveness trial to test if this novel patient-centered automated and customized mHealth-based SS intervention is effective and cost-effective enough to warrant future large-scale implementation into Uganda’s routine maternity care.

**Methods:**

We will physically recruit 824 pregnant women at <20 weeks of gestation living in Mbarara and Mitooma districts, southwestern Uganda, and randomize them (1:1) to receive standard of care or the Support-Moms app, with at least 2 of their identified social supporters. Our primary outcome will be the proportion of skilled births. Secondary outcomes will include number of ANC visits<strong>,</strong> institution-based delivery, mode of infant delivery, preterm birth, birth weight, SS, obstetric complications, and deaths (maternal, fetal, and newborn). We will assess other implementation, service, and client outcomes through study records, the mHealth platform, and questionnaires with all women in the intervention, their social supporters, health care providers (HCPs), and managers from participating facilities. We will conduct face-to-face in-depth exit interviews with 30 purposively selected intervention participants and 15 facility HCPs and managers to explore implementation strategies for scale-up. Annual maternity resource allocations, costs, number of ANC visits, and deliveries will be assessed from facility records up to 36 months after implementation. We will estimate incremental cost-effectiveness ratios concerning cost per additional HCP-led delivery, per death averted, and per quality-adjusted life year gained as cost-effectiveness measures.

**Results:**

This study was funded in September 2023. Ethics approval was obtained in February 2024, and actual data collection started in March 2024. As of January 2025, 75% (618/824) of all projected study participants provided consent and were recruited into the study. Participants are expected to be followed up until delivery, and 15% (124/824) have so far exited. Data analysis for the trial is expected to start as soon as the last participant exits from the study. The qualitative interviews will start in April 2025, and data will be analyzed and published as soon as data collection is done, which is expected in March 2027.

**Conclusions:**

We are testing the feasibility, acceptability, and cost-effectiveness of implementing Support-Moms into routine maternity care from individual and facility perspectives. We hypothesize that Support-Moms will be an effective and cost-effective strategy to improve maternity service use for women in rural Uganda and similar settings.

**Trial Registration:**

ClinicalTrials.gov NCT05940831; https://clinicaltrials.gov/study/NCT05940831

**International Registered Report Identifier (IRRID):**

DERR1-10.2196/67049

## Introduction

### Background

While maternal mortality has fallen over the last 20 years, an estimated 300,000 women die each year from preventable causes related to pregnancy and childbirth, and 99% of the deaths occur in low- and middle-income countries (LMICs) [1]. Over 99% of infant deaths also occur in LMICs [2,3]. Persistently high maternal mortality ratios (MMRs) in LMICs are partly attributed to challenges accessing care, with undiagnosed or poorly managed pregnancy-related complications from direct or indirect causes [1]. Recurrence risks of fatal episodes increase exponentially among women who survive previous fatal episodes unless preventive measures for early detection and monitoring throughout the perinatal period are adopted [2,4-6]. Antenatal care (ANC) prevents perinatal and maternal morbidity and mortality by early detection and treatment of prenatal complications and identifying women at high risk to ensure delivery in skilled settings [7-11]. ANC supports women, their families, and communities to navigate challenges at a critical time during pregnancy; debunks misconceptions; increases information transfer; and can motivate women to seek facility delivery and care [9]. The World Health Organization (WHO) and other authorities have called for urgent evaluation of adaptable and context-specific health solutions to promote ANC uptake and maternity services to reduce maternal and early childhood mortality and morbidity [9,11-14]. Identifying and scaling up interventions that improve access to and the use of available health care in pregnancy and childbirth has the potential to prevent 823,000 stillbirths, 1,145,000 neonatal deaths, and 166,000 maternal deaths annually in the 75 highest-burden countries [2,4-6].

Despite expanded capacity to increase the number of skilled birth attendants at community facilities, Ugandan women still have low rates of ANC use and skilled births, resulting in one of the highest MMR (189/100,000) and perinatal mortality rates (34 deaths/1000 births) in the world [15]. Women’s lack of information, social support (SS), financial independence for emotional and economic provisions, decision-making autonomy regarding childbirth, birth preparedness, and perceived need for maternity care services are important challenges to using available maternity services in these settings [1,16-19]. One analysis showed that women at risk of unskilled home births needed relevant and context-specific strategies to encourage ANC attendance and skilled delivery [20]. The high cost of MMR highlights the need for adaptable interventions that boost ANC and maternity services use to reduce MMR and early childhood deaths [12].

SS is an important pillar of health promotion that has been directly linked to health care–seeking behavior, infant care practices among mothers, and HIV care in sub-Saharan Africa (SSA) [21-25]. SS can mitigate structural and physical barriers to health care access at individual and societal levels, including facilitating self-efficacy to complete positive health behaviors [26-28]. Spouses, relatives, and friends have been the sources of SS among individuals living with HIV in SSA [23-25]. Community health workers (CHWs) can provide or promote additional SS during pregnancy, leading to better health outcomes [29,30]. Village health teams (VHTs), which comprise community members identified by their community who are trained on major health programs, improve timely care seeking for facility delivery [31-35]. VHTs have historically focused on the treatment of infectious diseases, such as malaria, pneumonia, and tuberculosis [35]. Their role as an additional resource for peripartum women has not been harnessed. Therefore, social network involvement not only addresses individual but also family- and societal- or community-level barriers to care in a setting with modest availability of health centers (HCs) providing needed services [17,25,36-38].

Mobile health (mHealth) interventions can be practical, effective, and scalable tools to improve maternal health care delivery and outcomes. Many SMS text messaging and other mHealth interventions can help individuals internalize risks and potential impact of various medical conditions as well as the needs and benefits of health services [11,13]. mHealth approaches can empower individuals to seek help, address specific health concerns, strengthen informed decision-making, and improve outcomes in the perinatal period [14,39]. Scheduled SMS reminders and telephone voice messaging approaches can enable people to increase control over their health by improving knowledge transfer, learning, and comprehension. These gains may improve the perceived need to use available services, especially when interventions are well directed and executed to provide accurate and relevant information on the promoted behavior [11,13,14,25,39-42]. Several studies have found that mobile phone–based messages can be motivational as a source of individual or family SS [43], cues to action [44], or to challenge societal negative beliefs [45]. Mobile phone interventions have also been shown to increase ANC attendance [46,47], institutional delivery [48,49] and vaccination rates (such as tetanus toxoid) [11,49].

mHealth interventions that specifically bolster SS can improve pregnancy experiences by decreasing anxiety and depression [50-53] and increasing perinatal bonding [52] and communication within social networks [53]. These benefits are mediated by promoting existing family structure and social networks, which in turn foster financial and emotional coping mechanisms to enable women to overcome socioeconomic and physical barriers to care, such as food insecurity, transportation, and provision of delegated service to overcome competing priorities [25,53-55]. Community and social network engagements toward mobilization of resources to enable health care access are practical, scalable, and sustainable approaches toward participatory health care financing and use [56]**.** Although SMS alone is a convenient and lower cost approach to support health care interventions with higher delivery success, the provision of multiple messaging options, such as voice messages and social networks involvement, has been crucial to extend reach beyond the individual literate personal phone owners in SSA [57,58].

Health awareness and motivating health care use are key elements of developing effective behavior change interventions [59]. However, mHealth interventions are not always effective in improving health care and use. Whereas the failure of impact has been attributed to a mismatch between the function, adaptability, and need for mHealth interventions in some settings [11,14], end-user designs that use iterative approaches in app development can improve health care service use [60]. In a formative study, we observed that knowledge gaps influenced women’s past and future decisions to not attend ANC and pursue unskilled home births [17,20]. Women were also largely dependent on their significant others for economic provisions, which, together with the existing gender and traditional norms, limited women’s ability and freedom to make family or health decisions to seek skilled care. Therefore, we developed an mHealth-based SS intervention (the Support-Moms intervention) using the health care use model by Andersen [61] that incorporates predisposing-, enabling-, and need-based factors to improve intervention uptake and service use. We then considered the framework by Bendixen et al [62] to personalize the information and tailor the system for our targeted end users. Our novel mHealth app was developed as part of a career development award (NIH-K43TW011004). This app or intervention was compatible with local regular mobile phone types, providing varying text and audio delivery mediums for individuals who were literate and who were not. In a randomized 3-arm pilot study (N=120) pregnant women who had not presented for ANC by their second trimester were equally randomized to receive (1) standard of care, which is the routine ANC information given at the maternity centers (control); (2) scheduled SMS audio messages from the final messaging prototype (scheduled messaging [SM]); and (3) SM, plus social supporter engagement through SMS (SS) [63].

We observed high intervention acceptability and feasibility, with >80% of women receiving ≥85% of intended messages within 1 hour. Over 95% of women found the app easy to use and compatible with their existing messaging programs; they also reported that the messages were useful and engaging and would strongly recommend the intervention to others. Nearly all women in the SS arm (39/40, 98%) had a skilled delivery compared to 78% (31/40) and 70% (28/40) of the women in the SM and control groups, respectively. All women whose social supporters were engaged on the app (SS arm: 40/40, 100%) attended ≥4 ANC visits, compared to 83% (33/40) and 50% (20/40) of the women in the SM and control groups, respectively. Fewer women (8/40, 20%) in the SS arm missed any visits due to the lack of transportation compared to 58% (23/40) and 68% (27/40) of the women in the SM and control groups, respectively. In addition, fewer maternal or fetal complications (3/40, 8%) were reported in the SS arm compared to 13% (5/40) and 25% (10/40) complications in the SM and control groups, respectively. Using the Duke-University of North Carolina (UNC) Functional Social Support scale [64], women in the SS arm reported improved SS (median 3.4, IQR 2.8-3.6) compared to 2.8 (IQR 2.6-3.2) and 2.4 (IQR 2.2-2.8) in the SM and control arms, respectively (score ranges from 1 to 4, and 4 indicates high levels of SS). In qualitative interviews, all women described the intervention as useful, actionable, and easy to use; tailored health information helped them to learn, internalize, and comprehend ANC and skilled delivery benefits, strengthening their informed decision-making as they were reportedly able to easily share and discuss information with their significant others, who in turn committed to providing them the needed support to prepare and seek help. Women also expressed that the involvement of their significant others within a friendly, trusted, and familiar environment helped them to mobilize needed support during pregnancy. Involving both health care providers (HCPs) and end users in characterizing, developing, and formulating the mHealth intervention allowed its tailoring to their preferences. Given the success in our pilot work where 78% (93/120) of the women used feature phones and promising preliminary efficacy data presented earlier [36,63], the next logical step was to assess the effectiveness, implementation, and scalability of such multiple messaging strategies to improve care access in SSA, where the contextual factors that drive successful interventions differ, but the public health impact of such interventions is likely to be the greatest [65].

### Objectives

We now propose a type 1 hybrid implementation-effectiveness trial to evaluate and implement this intervention into routine care. We will test the effectiveness of the intervention in a randomized controlled trial (aim 1). We will apply the implementation outcomes framework by Proctor et al [66] to evaluate implementation, service, and client outcomes and conduct in-depth interviews with users and key stakeholders to contextualize or clarify these outcomes as well as explore implementation strategies for future scale-up using the Consolidated Framework for Implementation Research (CFIR; aim 2). We will then assess the costs and cost-effectiveness of implementing the Support-Moms intervention into routine care (aim 3). We hypothesize that implementing Support-Moms will be an effective and cost-effective strategy to improve maternity service use.

## Methods

### Study Overview

**Figure 1:**
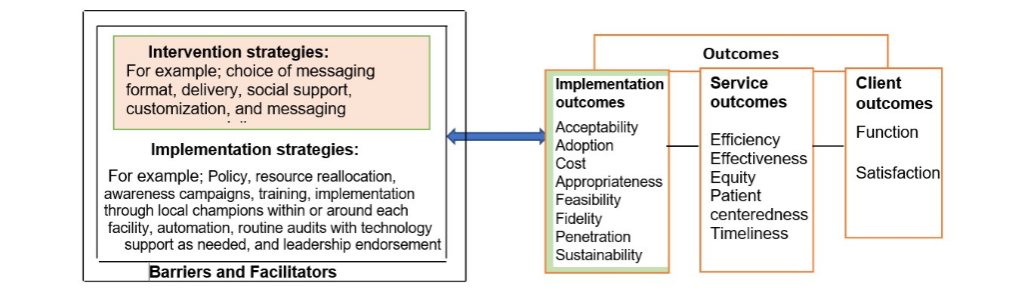
Modified conceptual framework by Proctor et al [66] for implementation and evaluation.

We propose to evaluate the effectiveness and implementation of the Support-Moms app into routine care. We hypothesize that Support-Moms will improve maternity service use and reduce the MMR when integrated into routine care. We will test the effectiveness of the Support-Moms intervention in a randomized controlled design (aim 1); evaluate intervention implementation using the implementation outcomes framework by Proctor et al [66], as outlined in Figure 1 (aim 2); and then refine implementation strategies for future scale-up using the CFIR, as outlined in subsequent sections [67]. We will assess the cost and cost-effectiveness of implementing this intervention into routine care and its implication for sustainability (aim 3). These outcomes will serve as indicators of implementation success or necessary preconditions for attaining desired service outcomes for women in rural, resource-limited settings. This will enable us to identify practical, context-specific, and actionable strategies for achieving optimal implementation effectiveness at a low cost. The intervention strategies were developed in our pilot work together with facility HCPs. In the cost-effectiveness analysis, if Support-Moms and standard of care are found to have equivalent effectiveness, we will conduct a cost-minimization analysis where only the cost of Support-Moms per participant will be estimated and reported (no incremental cost-effectiveness ratios [ICERs] will be calculated). This alternative approach would remain informative to the policy makers and stakeholders in maternity service use. Notably, simultaneous assessment is warranted given the (1) strong preliminary evidence, (2) relatively low investment needed for costing, and (3) overall efficiency of our proposed type 1 hybrid effectiveness-implementation trial approach.

### Study Setting

Uganda’s public health system is organized into 7 tiers with national and regional referral hospitals, general district hospitals, and 4 levels of community HCs. Staffing and available services vary across the 4 levels; HC3 carry out vaginal deliveries, whereas HC1 and HC2 serve as low-resource referral units. HC4s and hospitals conduct normal and cesarean deliveries and have ambulances and blood transfusion services [68]. Private HCPs operate in parallel to the public health system to provide maternal health care. Mbarara District is located approximately 270 km southwest of the capital, Kampala [69]. Mbarara District hosts a regional referral hospital that serves the southwestern region (Mitooma district inclusive); most deliveries are high risk [70]. Mitooma District borders Congo and is situated approximately 370 km southwest of the capital [71]. These 2 sites were selected for this research based on their geographic, sociocultural, and institutional diversity and high maternal mortality and morbidity data (Table 1 presents more details). Both districts have publicly funded and operated facilities with an active maternity care unit. Participants may be seen at any of the maternity sites in these 2 districts or other neighboring districts (Table 1), with recruitment and follow-up organized through CHWs. This consideration, plus the diversity of the settings and the study population, has potential for generalizability to similar settings. The local economy of these 2 districts is also largely based on subsistence agriculture, with both food and water insecurity being common [72,73]; ANC attendance of ≥4 visits is still at 58%, and the skilled facility delivery rate is approximately 70%. Maternity services, including delivery, are largely provided free of charge through public HCs.

**Table 1:** Mbarara and Mitooma district statistics 2019.

Characteristics	Mbarara, n	Mitooma, n
Total population	Approximately 250,000	Approximately 185,000
Annual registered ANC^a^ visits	31,200	18,350
Annual public facility deliveries	14,800	4450
MMR^b^ per 100,000 live births	328	412
Hospital availability	5 (4 are private)	0
Publicly funded HC4s^c^	2	1
Publicly funded HC3s	10	6
VHTs^d^	246	143
Other private facilities providing maternity services	34	12
Total HCPs^e^	253	104

^a^ANC: antenatal care.

^b^MMR: maternal mortality ratio.

^c^HC: health center.

^d^VHT: village health team.

^e^HCP: health care provider.

### Aim 1: Testing the Effectiveness of the Novel Support-Moms Intervention in a Randomized Controlled Trial

We will enroll a cohort of 824 adult pregnant individuals with gestational ages ≤20 weeks at enrollment (determined by last normal menstrual period or ultrasound scan where available). Consenting participants will be randomized 1:1 at enrollment to standard of care (Ministry of Health [MOH] guidelines–based routine care and information giving, n=412, 50%) versus the Support-Moms (intervention) group (n=412, 50%). We will identify, screen, and enroll people through the existing CHWs or VHT structure from areas within a 10 km radius of all publicly funded maternity centers across Mbarara and Mitooma districts, who have not yet presented for ANC by the beginning of their second trimester. We will power the study to test the superiority of the Support-Moms intervention for our primary effectiveness outcome: HCP-led skilled birth delivery. Secondary outcomes will include (1) number of ANC visits, (2) institution-based delivery, (3) SS, (4) mode of infant delivery, (5) all deaths (maternal, fetal, and newborn), (6) preterm birth, (7) birth weight, (8) breastfeeding, (9) completion of postnatal care, and (10) complications of pregnancy and childbirth (eg, obstructed labor, ruptured uterus, need for neonatal or maternal resuscitation or assisted ventilation, severe preeclampsia or eclampsia, postpartum hemorrhage, maternal or newborn sepsis, and other infections).

### Recruitment and Enrollment of Study Participants

We will include individuals who (1) are in the first trimester of pregnancy and have not yet presented for ANC, (2) reside in the catchment area of a study HC, (3) are emancipated minors and adults aged ≥18 years, (4) report access to a cell phone with reception in their home, (5) are able to identify at least 2 social supporters living within the study districts, and (6) are able to provide consent. Notably in our pilot study, >95% of screened individuals had access to a cell phone, and all were able to identify at least 2 social supporters living within their communities [63]. We will track the exclusion rates to inform generalizability. CHWs will notify study research assistants (RAs) about potentially eligible participants, who will then contact and seek written informed consent and assent for emancipated minors (ie, those aged <18 years and pregnant) before enrollment into the study. Participants will be asked to identify 2 individuals from their existing SS network with whom they have had stable, long-term relationships and believe they would be available to help them during the pregnancy and study follow-up period. Eligible social supporters will include spouses, relatives, CHWs, and friends [23-25] aged ≥18 years; who are aware that the study participant is pregnant; and who own a cell phone for personal use with reported reliable reception. Potential social supporters will be excluded from the study if they are unable to use SMS or are unwilling to receive SMS notifications, as this was identified as a barrier in the pilot study. In the pilot study, one of the eligible social supporters identified by women included a spouse (75%), friend (38%), sibling (10%), parent (53%), and a CHW (25%), and we expect a similar distribution in the trial. There will be no gender exclusion criteria for social supporters. We will emphasize the selection of an existing partner, who is aware of the pregnancy, as one of the social supporters. This was not a problem in our pilot study, as most women were able to suggest a partner as a potential social supporter, alongside a friend, sibling, parent, or CHW. A few partners were excluded because they did not own a cell phone for personal use (5/40, 13%) or were not aware of the pregnancy (5/40, 13%). All other eligible social supporters that were identified by participants in the social supporter arm (80/80, 100%) were successfully enrolled and completed study procedures. RAs will contact social supporters from the intervention arm within 2 weeks of the enrollment of pregnant women to confirm an active relationship at the time of their enrollment. Eligible social supporters will be offered an explanation of the study procedures and an opportunity to participate in informed consent. The study nurse will inform consenting social supporters about the objectives of ANC and skilled delivery as well as danger signs during pregnancy using standard MOH and WHO guidelines [9,74].

### Randomization

Before the study initiation, the study statistician will generate a randomization table, inaccessible by other study team members, and lock it and store it in the REDCap (Research Electronic Data Capture; Vanderbilt University) study database. Participants will be stratified according to district and HC level and randomly assigned to either intervention or control arms in a ratio of 1:1 in blocks of 10. RAs will be informed of the arm assignment by the REDCap module after consent and at the time of enrollment. Study participants in the control group will receive MOH guidelines–based routine care and information giving. The intervention group will receive the intervention described in the next section.

### Intervention Delivery and Components

The final messaging prototype that includes tailored SMS and audio health information (described earlier) will be delivered by the Support-Moms app developed through a partnership with iStreams-Uganda, an app development company based in Mbarara that developed the app, and with an existing mHealth platform [75]. The unique multimedia design allows women to be registered on the platform and be tracked throughout pregnancy and the postpartum period. Enrolled women receive automated and scheduled SMS text messages, reminders, and notifications about upcoming appointments as well as informational voice messages in their preferred language. The app includes a data collection platform and stores information submitted in real time directly from the participant’s phone, thus allowing managers to access up-to-date data on process measures (eg, automated SMS text messages sent and accessed) as well as intervention delivery and health outcomes. Fixed SMS data are stored in a secure cloud, which is Health Insurance Portability and Accountability Act (HIPAA) compliant. iStreams-Uganda works in partnership with Africa’s Talking, a platform that facilitates access to a telco infrastructure that uses automated SMS, voice, airtime, and other application programming interfaces—mechanisms tested and successfully used during our pilot study. This automated technology for SMS [23,25] and calls [36,63] has also been used for other studies in Uganda.

Both SMS and audio messages will be delivered at participants’ preferred time and day of the week for free to optimize intervention delivery. A weekly SMS reminder on the impending ANC appointment and expected date of delivery at their preferred time and day of the week, plus a day before the scheduled ANC visit, will be sent to study participants. Social supporters will receive weekly SMS notifications to motivate the pregnant women participants to be present for scheduled ANC visits during the pregnancy as well as for delivery. Notifications to the 2 preidentified social supporters will provide information about the upcoming ANC visits and delivery due date during the study follow-up period. ANC appointment dates will automatically be generated based on the provided LNMP and MOH guidelines [74] at enrollment. Social supporters will be able to personalize the SMS content at enrollment (the default message will be “This is your reminder to assist your friend [XXX] attend her upcoming ANC visit due soon”). They will also be advised to assist study participants with problems that may affect ANC attendance or facility delivery. The intervention is designed to build on existing supportive relationships of study participants within their communities. All women and their social supporters will receive all accredited messages included in this app for at least 4 months, including the standard routine care provided at the community maternity centers.

### Data Collection

Baseline participant characteristics will be collected from study participants from both arms as well as among the social supporters in the intervention arm physically (Table 2). Data collected through participant questionnaires will be conducted in the local languages, Runyankole and Rukiga. We will collect outcome data in two ways: (1) through medical record review of the routinely provided ANC cards, postnatal discharge forms (where available), and records at the relevant HCs and (2) through participant exit interviews 2 to 4 weeks following delivery to enhance data completion, particularly for people who did not deliver at a facility.

**Table 2:** Baseline questionnaire items for study participants and social supporters.

Topic		Details of measure
**Individual level**		
	Individual characteristics	Age, education, employment, socioeconomic status, marital status, religion, and self-efficacy^a^
	General and mental health^a^	We will assess psychological symptoms using validated Hopkins Symptoms Checklist for depression and anxiety [76,77].
	Alcohol or substance use	We will assess alcohol use using the 3-item consumption subset of the AUDIT-C^b^ [78,79] due to its association with adherence and health outcomes.
	Reproductive history	Gravidity, parity, gestational age, prenatal and antepartum high-risk morbidities, and NCDs^c^
	Pregnancy and childbirth perceptions	Health beliefs, knowledge and risk awareness, need for skilled delivery, and childbirth practices
**Relationship level**		
	Reproductive goals and motivation	We will adopt the 6 items used in Uganda to assess personal and partner pregnancy desires [80-82], In total, 18 questions or statements reflect 6 parenthood motives [83].
	Relationship power and gender-based violence^a^	We will assess gender-based violence [84] and relationship power [85-87] given its relationship with home births in Uganda [17].
	Social support^a^	We will adopt and measure social support using a version of the Duke-UNC^d^ Functional Social Support Scale [26], a tool that has been widely used in Uganda [64].
**Community level**		
	Service availability^a^	Distance to the nearest health facility, availability of midwives, history of home or facility birth, community support for alternative birthing choices, and relationships with HCPs^e^
**Societal level**		
	General health and food insecurity^a^	We will assess the general health of women, including diagnosed NCDs, and measure food insecurity using the HFIAS^f^ [88]
	Societal norms^a^	Beliefs about pregnancy, childbirth, birth order, twin delivery, facility delivery, and fatality
	Quality of life^a^	Improved Short Form-6 Dimension version 2 survey by Brazier et al [89] to assess the quality of life

^a^Collected at exit interviews.

^b^AUDIT-C: Alcohol Use Disorders Identification Test-Consumption.

^c^NCD: noncommunicable disease.

^d^UNC: University of North Carolina.

^e^HCP: health care provider.

^f^HFIAS: Household Food Insecurity Access Scale.

To additionally reduce the risk of missing data, for participants who cannot be contacted, we will conduct home visits and interview the next of kin for those who are lost from observation or who die during the study period. These survey data will include the date and location of the birth; whether there was a skilled birth attendant present; mode of delivery (ie, vaginal vs cesarean delivery); birth outcome, including preterm birth, maternal, fetal, and newborn deaths, and any other complications of the birth (eg, obstructed labor, ruptured uterus, need for neonatal or maternal resuscitation, severe preeclampsia or eclampsia, postpartum hemorrhage, maternal or newborn sepsis, and other infections); weight and height of the newborn; number of ANC visits completed; use of breastfeeding; and attendance at postnatal care. Finally, we will administer the Duke-UNC Functional Social Support Questionnaire to measure reported SS received by women during pregnancy and childbirth.

### Aim 1: Analysis Plan and Sample Size Calculations

We will first summarize health-related and sociodemographic data between arms. For our primary effectiveness outcome, HCP-led skilled birth delivery, we will fit a multivariable logistic regression model, with study arm as the predictor of interest, and age, high-risk pregnancy, and health facility at enrollment as a priori additional variables in the model, due to their strong association with the selected outcome [70,90,91]. In our primary intention-to-treat model, we will consider women with missing outcome data, after home visits and next of kin interviews, as presumed to have not received skilled birth (ie, there will be no missing outcome data in our primary analysis). In sensitivity analyses, we will (1) repeat the analysis after excluding women with missing outcome data and (2) include additional potential confounders in the model that may have persisted despite individual randomization (eg, number of previous deliveries at a facility, socioeconomic status, distance to facility, history of facility delivery, food insecurity, alcohol use, and depression). Although not designed to detect a difference, we will also explore additional secondary outcomes, including (1) number of ANC visits completed, (2) mode of infant delivery, (3) institution-based delivery, (4) presence of one or more birth complications, (5) child mortality, (6) maternal mortality, (7) preterm birth, (8) birth weight, (9) completion of postnatal care, (10) SS, and (11) initiation of breastfeeding. Both our pilot data and other similar studies estimate that 70% of the women in Uganda deliver with a skilled attendant [15,48,49]. Finally, we will explore the role of SS as a moderating effect of the intervention through a prespecified stratified analysis among women in the upper versus lower half of SS in the cohort, as measured by the Duke-UNC Functional Social Support Scale [64]. To test our primary effectiveness hypothesis, allowing for a 2-sided type I error of 5%, 90% power, and assuming a 5% loss to follow-up, we will require 824 participants to detect a 10% difference in HCP-led skilled birth delivery between arms. Data analysis will be conducted using Stata (version 17; StataCorp LLC). The findings will be presented as descriptive statistics, scatter plots, and graphs; statistical significance will be considered at *P*≤.05. While we will ensure completed data are collected through timely cleaning and REDCap prompts, we will still be able to detect the same effect in the primary outcome with the power of 85% in the unlikely event that we lost up to 18% of the records due to missing data.

### Aim 2: Evaluating Intervention Implementation

We will evaluate intervention implementation using the framework by Proctor et al [66] (Figure 1) and plan for future scale-up per the CFIR [67] (Table 3). While the effect of the Support-Moms intervention on HCP-led skilled birth delivery, ANC attendance, and other secondary outcomes in aim 1 is critical, the translation of its potential benefit into routine care impact requires understanding the implementation process. We chose the evaluative framework by Proctor et al [66] because it consists of essential implementation science outcomes with attention to both services and clients, which will be critical for uptake and long-term use of Support-Moms in routine care (Figure 1 and Table 4). Then, we will consider and refine implementation strategies for future scale-up using the CFIR as a determinant implementation science framework (Table 3) [67].

**Table 3:** Consolidated Framework for Implementation Research (CFIR) constructs that will guide data collection on intervention challenges, facilitators, and potential strategies by care users, health care providers (HCPs), and payers and managers.

CFIR construct	Interview topic	Possible questions to elicit implementation strategies from users and implementers
Intervention characteristics	Intervention-setting fitness, automation, and auditing	On the basis of the reported or presented acceptability, effectiveness, patient centeredness, satisfaction, and function outcomes, how can the intervention be improved for increased implementation?
Outer setting	Existing policy, resources, and MOH^a^ willingness and capacity to support increased demand for services and adopt the intervention	On the basis of the presented adoption, penetration, and other outcomes, as well as existing policies and resource commitments, what rollout strategies will be most effective in overseeing intervention implementation?
Inner setting	Facility adaptive reserve, leadership endorsement, and resource reallocation	On the basis of the identified cost-effectiveness, how can existing resources be reallocated to promote intervention uptake?
Individual characteristics	Patient centeredness, support, and community referrals	On the basis of acceptability, satisfaction, and function, what potential support will be needed for individuals using Support-Moms to improve uptake, enthusiasm, and retention?
Implementation process	Experience of HCPs on app enrollment, patient interaction, and increased demand and implementer’s intention to “try,” budgeting, sustainability, timing, execution, and scale-up	Explore implementers’ support and satisfaction of the app to improve service use; approaches to publicizing and dissemination; engaging CHWs^b^, focal HCPs, and social supporters as champions; long-term funding, potential impact; and “leading” or “lagging” indicators of the implementation success

^a^MOH: Ministry of Health.

^b^CHW: community health worker.

**Table 4:** Application of the framework by Proctor et al [66] to evaluate implementation, service, and client outcomes.

Outcomes and domain		Specific intervention measures	Data source
**Implementation outcomes**			
	Acceptability	Reported ease of use and performance expectancy, effort expectancy, social influence, facilitating conditions, self-efficacy, and behavioral intention to use the app in the future Overall user acceptability per the tool by Weiner et al [92] Qualitative: For example, participants: How was it for you to use these SMS, messaging, or calls? and HCP^a^: How was it for you using the messaging app?	Exit interviews Exit questionnaire
	Adoption	Initiation and use of the app over time Percentage of eligible and participating social supporters	mHealth^b^ platform Study records
	Appropriateness	Relevance (for setting) and compatibility Overall appropriateness with the tool by Weiner et al [92] for both HCPs and end users Qualitative: For example, participants: What happened when you received SMS or voice calls? and HCP: What happened or what did you observe when you enrolled people on the app?	Exit interviews Exit questionnaire
	Cost	Refer to aim 3: Evaluating the Cost and Cost-Effectiveness of Implementing the Support-Moms Intervention Into Routine Care and Its Implication for Sustainability section	Refer to aim 3
	Feasibility	Percentage of users willing to participate; percentage of women, spouses, and social supporters meeting eligibility criteria; recruitment or participating rates, and reason for not participating. We will use the tool by Weiner et al [92] to measure feasibility for both HCPs and end users.	mHealth platform Study records Exit questionnaire
	Fidelity	Percentage of HCs^c^ with capacity and integrity to deliver intended service (ANC^d^, skilled deliveries, and admissions), percentage of accessible cell phones, and percentage of messages automatically sent out Percentage of SMS or voice calls received by the participant over anticipated per protocol Percentage of network, dead battery, phone losses, and phone functionality issues encountered	Study records mHealth platform Facility audits
	Penetration	Number and type of HCs and HCPs engaging with the app Percentage of eligible participants and social supporters enrolled	Facility audits Study records
	Sustainability	Use of the app over time, social supporter engagement over time, and user retention Percentage of participants lost to follow-up and percentage of additional staff needed Qualitative: All, for example, what challenges did you experience or face while using this app?	Facility audits Study records Exit interviews
**Service outcomes**			
	Efficiency	Time spent on enrolling participants on the app, time spent on deliveries, timely delivery of needed supplementary or reference information, cost of delivery Qualitative: All, for example, what do you think about this intervention?	mHealth platform Exit interviews
	Effectiveness	Refer to aim 1	Refer to aim 1
	Equity	App use by facility type, participant type, and demographics	Study records
**Patient centeredness: accomplished during the formative and pilot work [36,60]**			
	Timeliness	Perceived impact on ANC attendance and skilled deliveries Qualitative: How was your experience attending ANC and preparing for birth and delivery?	Exit interviews
**Client outcomes**			
	Function	Perceived quality, impact on maternity care, use, and life (survey by Brazier et al [89]) Qualitative: How did this intervention help you in your pregnancy or work as an HCP?	Exit questionnaire Exit interviews
	Satisfaction	Satisfaction with intervention content, delivery, and credibility (Client Satisfaction Questionnaire) [93] for both HCPs and end users Qualitative: What concerns do you have about using this technology to support you?	Exit questionnaire Exit interview

^a^HCP: health care provider.

^b^mHealth: mobile health.

^c^HC: health center.

^d^ANC: antenatal care.

### Data Collection

#### Implementation Metrics

A trained RA will administer interviewer-led questionnaires at the trial exit (Table 4 presents outcomes and data sources) to (1) all intervention arm postpartum women, (2) all intervention arm social supporters, (3) all HCPs from participating facilities who enroll and deliver participants within the study sites, and (4) MOH key stakeholders and managers expected to inform rollout and adoption by the MOH. We will use a standardized checklist to conduct facility audits during implementation to document ANC visits and deliveries registered; maternity admission data; maternal mortality; disease cases managed; prescription data; laboratory data; and resource allocations at baseline, 12, 24, and 36 months following the implementation of the intervention for all study sites. A facility inventory will be done to inform our understanding of baseline conditions and set up for maternity and reproductive health services at the hospital and HC4 and HC3 public maternity centers.

We will use quantitative data on reported acceptability, appropriateness, effectiveness, function, and satisfaction to purposively select a subset of up to 15 postpartum individuals for exit in-depth interviews (or until saturation is met [94]), 15 social supporters, and up to 10 HCPs (approximately 2 for each facility level or district; refer to Table 1) involved in participant app enrollment and facility deliveries to clarify or contextualize observed outcomes based on the framework by Proctor et al [66] as outlined in Table 4. We will ask participants to describe actual events and experiences wherever possible (eg, for postpartum individuals, what worked well or poorly with receiving the messages and the challenges experienced during the study) to not only ensure coverage of specific areas but also allow unanticipated themes to emerge. We will further explore the feasibility, appropriateness, acceptability, patient centeredness, and sustainability of involving social networks in this intervention, as well as relationship dynamics, which have been shown to influence social supporter interventions [23-25] (eg, the social supporter’s specific role, their relationship throughout pregnancy, routine communication, the type of voluntary and requested SS given or received during pregnancy toward improving her experience, ANC visits, birth preparedness, childbirth, and the app-related challenges and opportunities). We will schedule these interviews between 4 and 6 weeks post partum. HCPs will also be interviewed at the end of the study to clarify potential opportunities and problems that were experienced with the intervention and its delivery. These will facilitate appropriate conclusions about effectiveness and implementation success.

#### Implementation Strategy Development

Using CFIR, we will develop guides to further interview these postpartum individuals, social supporters, and HCPs on the intervention, individual, and inner settings to inform our implementation strategies and optimize intervention delivery (Table 3). We will develop some initial implementation strategies based on these findings and the literature (eg, awareness campaigns in the community and dissemination or publicizing the app, implementation in facilities through local champions within or around each facility to maintain enrollments, training, choice of messaging format, automation, and routine audits with technical support as needed). At the end of the implementation period, we will present data on outcomes and cost-effectiveness of the framework by Proctor et al [66] (refer to aim 3), along with the preliminary implementation strategies to the facility, district, and national MOH managers and stakeholders who have key roles in budgeting and policy or service implementation and are expected to evaluate or endorse the app. Using CFIR-informed interview guides (Table 3), we will interview 5 to 10 of these key MOH managers and stakeholders for feedback and refine our initial implementation strategies for testing in a subsequent study. All qualitative interviews will be audiotaped with the participant’s permission and transcribed verbatim. RAs will be trained on the interview guides. All HCP and MOH interview guides will be piloted with 3 staff managers at Mbarara Hospital to ensure optimization, comprehension, and appropriateness.

### Intervention Fidelity

Intervention fidelity plays a key role in assessing intervention effectiveness [95,96]. An RA will ensure participants know how to use the phone to retrieve information. CHWs and HCPs from the targeted public HCs will be trained to enroll participants onto the app, with technical support from the study research teams, led by ECA and GRM. We will measure the 3 elements of implementation fidelity as outlined in Table 4. Notably, 13% of the women enrolled in our pilot study missed some app messages because of lost phones or phone functionality issues. To minimize dead battery and charging issues, solar chargers will be given to study participants to charge their phones as needed during enrollment. These chargers are readily available and inexpensive, and we will be tracking their cost. Phone losses will be assessed on a case-by-case basis, replaced sparingly, and costs will be determined accordingly. Outgoing SMS and voice messages will be monitored daily by the data management team. The times and lengths of individual outgoing calls and engagements will be recorded and transmitted to the server. Message deliveries during periods of inadequate cellular reception will be stored for later transmission. Although 2-way messaging has been found to be useful in other settings [11,14], our key factors in this proposal are to provide scheduled, targeted information and catalyze SS for women seeking maternity care, a mechanism that showed promising results in our pilot preliminary data. Moreover, 2-way messaging creates a burden on the health system that may not be sustainable. However, we will assess the need for this type of feature in the exit interviews for further exploration. In addition, contact numbers of the VHT and CHWs attached to the neighboring public HC will be provided to address any questions, referrals, or emergencies that may arise. SMS and voice call delivery or reception will be considered as proxies for accessing information to alter existing predisposing factors (such as negative health beliefs and low awareness) that could enable and improve perceived need to seek care with the help of available social networks, factors that will be assessed during exit interviews (Table 2 presents more details).

All quantitative data will be collected using a web-based database that will be developed in REDCap to improve data completeness, management, and quality control monitoring. Errors or out-of-range entries are reported immediately on the website so the original interviewer can reconcile the problem in a timely manner. Data entry verification will include algorithms that automatically check completed forms for missing, out-of-range, or inconsistent values before a form can be saved on the website.

### Aim 2: Quantitative Analysis Plan

We will summarize implementation outcomes for Support-Moms users and implementers using descriptive statistics. Success in the implementation survey data will be identified qualitatively and by the top tertile of relevant scales (eg, acceptability, feasibility, satisfaction, and appropriateness). We will explore similarities and differences across HCs and districts over time as well as potential associations between implementation outcomes and effectiveness at HCs and district levels. We will summarize all findings and present them through a Delphi process or technique [97], with a final meeting involving key MOH managers and stakeholders. We will describe the ranked implementation strategies selected by app users and key MOH managers and stakeholders after the dissemination process.

### Aim 2: Qualitative Analysis Plan

In-depth interviews will be digitally recorded and transcribed. The first set of exit interviews will be conducted to understand participants’ or stakeholders’ experiences and perspectives of the Support-Moms intervention and clarify the implementation, service, and client outcomes outlined in Table 4. The goal of the CFIR-informed interviews will be to refine and inform implementation strategies for integrating the Support-Moms intervention into routine maternity care. Qualitative analysis will be inductive [98], and categories will be derived from the different study textile participants, HCPs, and MOH manager and stakeholder interviews. These responses will be transcribed into English, if needed, and coded using NVivo (version 13; Lumivero). Data analysis will be jointly performed. The study coordinator and research fellow will double code 5 sampled transcripts from each category and, together with the principal investigator, resolve any coding disagreements to ensure consistency in the codebook. Dyadic analysis will also be performed between pregnant individuals and their social supporters. Categories will then be developed and presented with illustrative quotes from data to explain experiences, challenges with the intervention, and recommendations to improve its implementation into routine maternity care.

### Aim 3: Evaluating the Cost and Cost-Effectiveness of Implementing the Support-Moms Intervention Into Routine Care and Its Implication for Sustainability

The incremental cost and the cost-effectiveness of the Support-Moms intervention or program will be estimated from HCP and health system and societal perspectives to guide the decision makers on continuation, incorporation, integration, sustained use, and routinization—a method that has previously been used in a Ugandan context [99]. We will measure and record the cost of developing and implementing the intervention (program costs), the cost to HCPs from increasing demand for or use of services, and costs to users (intervention participants and their social supporters). The cost of developing and delivering the intervention will be estimated in consultation with the app developers, data from the pilot study, the maintenance team, time and motion studies conducted at representative sites over a 2-week period, as well as administrative records during implementation. The costs of maternity service use to access care will be collected from all aim 1 participants and HCPs at exit; all intervention users and HCPs will provide the cost of care seeking and intervention involvement. We will identify comprehensive tasks required by both users (eg, time used to seek care) and HCPs (eg, training and staff time) and quantify public-sector resource use during the use and provision of the HCP-led service during the 3 years of implementation. Routine and additional public-sector unit costs will be collected from the health management information system [100] and administrative records at the facility and district levels, MOH, and other safe motherhood implementing partners. Direct and indirect costs to intervention users and HCPs because of involvement in the intervention (such as trainings, time used to seek care, and time used by HCPs to enroll users on the app) will be collected from participant exit interviews and administration records. The costs of providing user phones and solar chargers will be explored.

### Cost-Effectiveness Analysis

Cost analysis will include estimating program costs, costs to HCPs, and costs to users as described above. We will also develop a decision tree model to assess the potential impact of economic, clinical, or health outcomes of the Support-Moms intervention against routine care [101,102]. The model will incorporate cost items, relevant clinical probabilities, and case outcomes, allowing a cost-effectiveness evaluation. We will combine the costs and outcomes for each branch of the tree using branch possibilities to simulate the expected costs and outcomes of the intervention and routine care. We will hypothesize service users using routine maternity services or enrolled on the Support-Moms app (per trial arm) as they pass through different health states over time and within acute and chronic health states of 3 key maternal morbidities of sepsis, postpartum hemorrhage, and hypertensive disorders [70,103-105]. Our cost-effectiveness evaluation will account for the outcomes for both the mother and the infant throughout their lifetime at the annual discounting rate of 3% [106]. The clinical and cost outcomes for the infant (eg, low birth weight and stillbirth or intrauterine fetal deaths) will be incorporated in the decision tree. We will use 1-way and probabilistic sensitivity analyses to quantify the confidence level or robustness in this model analysis output in relation to the outputs and the payer’s willingness to pay thresholds [101,107]. We will estimate ICERs in terms of cost per additional HCP-led skilled birth delivery and per death averted. In addition, we will estimate ICERs per disability-adjusted life years averted and quality-adjusted life years (QALYs) gained (QALYs derived from the collected SF-6Dv2 data) as the main summary measures of cost-effectiveness [108] in line with the country’s gross domestic product per capita (GDPpc) [109], to assess the value for money of adopting or providing the Support-Moms app long-term compared to routine care. If ICERs per QALY or per disability-adjusted life years <300% of GDPpc, Support-Moms will be deemed cost-effective, highly cost-effective for ICERs <100% of GDPpc, and not cost-effective otherwise. The decision tree and cost-effectiveness analyses will be programmed in TreeAge (TreeAge Software, LLC).

### Ethical Considerations

The ethics approval was obtained from the Mbarara University of Science and Technology Research Ethics Committee (MUST-2022-631) and Uganda National Council for Science and Technology (HS3366ES). Study site administrative permission was obtained from the Mbarara district health officer, Mbarara City Health Officer, Mitoma District health officer, and the director of clinical services at the MOH. We are continually seeking and obtaining written informed consent from all study participants before enrolling in the study. The participants can withdraw from the study at any time. Participant data are anonymized at all times. The study was registered at ClinicalTrials.gov (NTC05940831). The research outcomes from this study will be published in international peer-reviewed journals and presented to the Ugandan MOH as policy briefs and at selected national and international conferences.

We are making firm attempts to adequately explain study purpose, schedule, expectations at the time of enrollment, and data collection and continually updating residence and phone details at each visit to minimize loss to follow-up. We are using appropriate means of contact based on participant preference and information on the best telephone network for the time of the day to telephone or send text messages, with emphasis that participation in this study is voluntary. However, reasons for decline or withdrawal from the study are being sought and documented. Participants are reimbursed for their study visits; a small compensation for their transport worth approximately UGX 20,000 (US $5.50) altogether is given at the end of each of the 2 planned baseline and exit interviews. Participating HCPs are reimbursed with US $5 as compensation for their time after every interview. The estimated time needed for interviews and other study procedures per visit is approximately 1.5 hours.

## Results

This study was funded in September 2023. Ethics approval was obtained in February 2024, and actual data collection started in March 2024. As of January 2025, 75% (618/824) of all projected study participants have consented and been recruited into the study. Participants are expected to be followed up until delivery, and 15% (124/824) of the participants have exited to date. Data analysis for the trial is expected to start as soon as the last participant exits (expected in March 2026). The qualitative interviews will start in April 2025, and data will be analyzed and published as soon as data collection is done. Data collection is expected to be completed by March 2027.

We are currently recruiting at least 60 women or individuals and their social supporters per month. We have registered no loss to follow-up so far.

## Discussion

We are targeting to recruit 824 pregnant women or individuals who have not yet presented for ANC by their second trimester, residing in Mitooma and Mbarara districts, with self-reported access to a cell phone with reception in their home for personal use, and are able to identify at least 2 social supporters living within the study districts. Uganda has approximately 30 million people who access mobile phones (most adult Ugandans), and 71% of users are connected using a basic feature phone [110]. Women or individuals are being identified by CHWs and VHTs, who then notify the study RAs to contact and seek written informed consent before enrollment into the study. Eligible social supporters who own a cell phone for personal use with reliable reception and know the study participant is pregnant be asked to provide consent and will be recruited within the first 2 weeks preceding enrollment of the pregnant women or individuals to ensure an ongoing relationship at the time of their enrollment. Adult HCPs who conduct deliveries within the study sites and MOH facility managers and stakeholders who participate in budgeting, procurements, or funding for facilities are being identified and offered enrollment into the study.

We developed an intervention aimed at improving communication of targeted, health-related information, motivating and mobilizing SS for pregnant women to use maternity services in rural southwestern Uganda (Support-Moms) [36]. Through an iterative approach, we (1) identified preferred key ANC topics from stakeholder interviews with 30 women and 5 HCPs and characterized a preferred messaging intervention; (2) developed content for SMS text and audio messages with 4 medical experts based on identified topics; (3) designed an app prototype through partnership with an mHealth development company (iStreams); and (4) pilot-tested the prototype and sought user experiences and feedback to refine the intervention through 3 different sets of 10 iterative exit interviews, 2 focus group discussions, and 5 cognitive interviews.

We are currently conducting a type 1 hybrid effectiveness-implementation trial [111] to test if this novel patient-centered mHealth-based SS intervention is effective and cost-effective if implemented into routine care from individual and facility perspectives. We will simultaneously assess other implementation, service, and client outcomes per the framework by Proctor et al [66] and refine implementation strategies for future scale-up using the CFIR (individuals, intervention, inner and outer settings, and process). We hypothesize that this intervention will be an effective and cost-effective strategy to improve maternity service use for women in rural Uganda and similar settings. Data collection is underway. Our results will present the functionality of our mHealth intervention, its ability to stimulate and encourage routine health care use, and improve maternal-fetal health outcomes among all rural women, including those with limited education. The results of our work will be usable for other groups designing similar interventions to promote perinatal health in resource-poor settings. Results of this study will also provide requisite data for maternal health policy change and lay the groundwork for evaluation for a regional implementation of the intervention.

Notably, our study will have some strengths. Unlike in many studies, we used conceptual frameworks to characterize and develop patient-centered content and design aimed at making findings more relevant and generalizable to rural communities where the impact of such interventions is likely to be the greatest. This approach is often lacking in mHealth development, most of which is often led by developers and investigators, with limited input from end users [11,14,112]. While many mHealth interventions have been developed in Uganda [113-115], very few have been in the reproductive health field [114,116], and fewer have been evaluated at scale in the public sector [115,117]. Therefore, our study will be among the first ones to test mobile maternal health apps in a randomized controlled trial in Uganda, concurrently assessing effectiveness and other implementation metrics, information that is critical for guiding ultimate use and integration of this intervention in routine care. In this study, we are studying a high-risk population, in which <70% of women deliver with a skilled attendant [15], <58% attend at least 4 ANC visits (of the 8 recommended by the WHO), and thus are likely to benefit from this intervention. We believe that our grounded approach, using appropriate implementation science models and partnering with key regional stakeholders to evaluate an intervention in a rural low-resource setting, will enhance the likelihood of uptake, adoption, and integration into routine care.

We are leveraging existing CHWs, social networks, and resources to encourage uptake, retention, and adoption within a community that largely depends on family and community networks to thrive [42]. This approach is hypothesized to improve pregnancy experiences, partner involvement, support, communication, and mental health during and after pregnancy, ultimately offsetting the downstream cost of avoidable maternal morbidity and mortality [50-53]. We are also building on our pilot, promising preliminary data, and it will provide vital evidence about effectiveness, uptake, and sustained use of this tailored mHealth approach designed to address common individual, family, and community or societal barriers to health care use in Uganda.

We are building on our experience from our previous work done within a typical public health facility setting to recruit and follow up participants. Many people in Uganda move frequently in search of stable work or new settlements, including pregnant individuals [25]. In addition, some change or lose their mobile phones or phones could be inaccessible at times due to network issues. We are using our previous clinical research experience in conducting similar trials to maximize the feasibility of our mHealth intervention and retention in care. SMS distribution is controlled, and women are routinely scheduled for ANC randomly and independently. Consequently, the risk of contamination (eg, discussing and sharing information) at the facility level is minimized. To minimize potential contamination in the community, we are making an effort to clearly explain the study procedures to CHWs or HCPs and intervention women who may learn about different arm allocations through casual conversations, an approach that worked well during our pilot. We have developed and administered a quality control checklist for a few randomly selected control participants so far to assess contamination between arms every 3 months.

## Appendix

Multimedia Appendix 1 Peer review report from the SIHH - Science of Implementation in Health and Healthcare Study Section, Eunice Kennedy Shriver National Institute of Child Health and Human Development (National Institutes of Health, USA).

## Abbreviations

ANC: antenatal care
CFIR: Consolidated Framework for Implementation Research
CHW: community health worker
GDPpc: gross domestic product per capita
HC: health center
HCP: health care provider
HIPAA: Health Insurance Portability and Accountability Act
ICER: incremental cost-effectiveness ratio
LMICs: low- and middle-income countries
mHealth: mobile health
MMR: maternal mortality ratio
MOH: Ministry of Health
QALY: quality-adjusted life year
RA: research assistant
REDCap: Research Electronic Data Capture
SM: scheduled messaging
SS: social support
SSA: sub-Saharan Africa
UNC: University of North Carolina
VHT: village health team
WHO: World Health Organization

## Data Availability

The datasets generated or analyzed during this study will also be available from the corresponding author on reasonable request.
