# Supplementary material for: Integration of a Patient-Centered mHealth Intervention (Support-Moms) Into Routine Antenatal Care to Improve Maternal Health Among Pregnant Women in Southwestern Uganda: Protocol for a Randomized Controlled Trial
Source: JMIR Res Protoc. 2025 Mar 19;14:e67049. doi: 10.2196/67049 (PMC11966083; doi:10.2196/67049)
Supplement: Multimedia Appendix 1 [file resprot_v14i1e67049_app1.pdf]

1R01HD111692-01A1 Atukunda, Esther

EARLY STAGE INVESTIGATOR
NEW INVESTIGATOR

RESUME AND SUMMARY OF DISCUSSION: The resubmitted study aims to assess the effectiveness and implementation of the Support-Moms intervention in routine practice to improve maternity service utilization in rural Uganda, a region with high maternal and perinatal mortality. The significance of the study is high in that if successful, it will have important contributions to improving utilization of maternity care services and improve maternal and child health outcomes in low-resource settings. The study is built upon on rigorous preliminary data that demonstrate high feasibility, acceptability, and uptake of the mHealth intervention. The research will be conducted by an impressive team led by a principal investigator who has a strong track record in mHealth inventions and research experience in Uganda. The team members have collaborated before, and present complementary expertise in reproductive health, implementation science, and health economics. The team's extensive research experience and network in Uganda is evident. The study is innovative in its use of a potentially sustainable intervention platform to deliver a multilevel intervention through lost-cost technology. Grounded in appropriate implementation frameworks, the approach is rigorous with many strengths including solid pilot work, well defined effectiveness outcomes, well thought-out mixed methods, robust quantitative and qualitative data analysis plan, and strong attention to sustainability. The application is highly responsive to the previous review with all issues adequately addressed. Overall, the committee is highly enthusiastic about this study and believe it is a very important project and the results will have a high impact on advancing interventions to improve maternal and child health outcomes in Uganda and other similar settings.

DESCRIPTION (provided by applicant): Antenatal care (ANC) and skilled births are mainstays of preventing maternal and perinatal morbidity and mortality. Despite expanded availability of skilled birth attendants and referral health systems, Ugandan women have low ANC use and skilled births, resulting in one of the highest maternal mortality ratios and perinatal mortality rates in the world. Mobile health (mHealth) interventions can support individuals to internalize risks, need, and benefits of health services with high intervention delivery success. Provision of multiple messaging approaches—such as scheduled SMS, telephone voice messages and social support engagement—can empower individuals to seek and access care, and improve health outcomes. However, despite successes in pilot studies, there is little data on effectiveness, appropriateness, feasibility, fidelity and incremental costs needed to adopt, or scale up such strategies in sub–Saharan Africa, where the public health impact of such interventions is likely to be the greatest. As part of a K43 career development award (PI Atukunda), we used behavioral frameworks to develop a user-centered mHealth-based, audio-SMS messaging application to support pregnant individuals to use maternity care services in rural Uganda (Support-Moms app). The app shared health-related information and engaged social support networks via scheduled SMS/audio reminders and upcoming ANC appointment notifications. In a randomized 3-arm pilot study (n=120) comparing standard of care (SOC), scheduled messaging (SM), and scheduled messaging plus social supporter engagement (SS), we observed high intervention uptake, acceptability, and feasibility. All women whose social supporters were engaged on the app attended ≥4 ANC visits, compared to 83% and 50% of women receiving only messages and SOC, respectively. Nearly all women in the SS arm (98%) had a skilled delivery compared to 78% and 70% in SM and SOC groups, respectively. We now propose a type 1 hybrid implementation-effectiveness trial to evaluate and implement the Support-Moms intervention into routine care. We will test the effectiveness of the intervention in a randomized controlled trial (N=824); our primary outcome will be the proportion of healthcare provider-led skilled births (Aim 1). We will apply Proctor's implementation outcomes

framework to evaluate implementation, service and client outcomes, and conduct in-depth interviews with users and key stakeholders to contextualize/clarify these outcomes, and explore implementation strategies for future scale-up using the Consolidated Framework for Implementation Research (CFIR) (Aim 2). We will assess costs and cost-effectiveness of implementing Support-Moms into routine care (Aim 3). We hypothesize that Support-Moms will be an effective and cost-effective strategy to improve maternity service utilization. This proposal directly responds to NIH and NICHD priorities described in PAR-22-105 and PAR-22-132 to reduce preventable causes of maternal deaths and improve health for women during and after pregnancy.

PUBLIC HEALTH RELEVANCE: High maternal mortality is a major public health problem in many settings. Because of low antenatal care (ANC) and skilled birth usage, Ugandan women and their children suffer from high maternal and perinatal mortality. We developed a promising intervention (Support-Moms app) that shares targeted health information, and engages social support networks through scheduled reminders to help support pregnant women to utilize maternity services in rural Uganda. We now propose to test and implement the Support- Moms intervention and hypothesize that Support-Moms will be feasible and cost-effective in improving utilization of available maternity care services, and ultimately reduce maternal and perinatal mortality.

CRITIQUE 1

Significance: 1
Investigator(s): 1
Innovation: 2
Approach: 1
Environment: 1

Overall Impact: This R01 application aims to evaluate the effect of the Support-Moms intervention and optimize its implementation in routine practice to improve maternity service utilization, and maternal-fetal outcomes among rural, low literacy, and underserved populations. This R01 is a revised application incorporating NIH reviewers' comments. The Support-Moms was developed under a prior compelling K and the pilot data about its potential effectiveness and acceptability and feasibility is extremely compelling. The Support-Moms app shares targeted health information, and engages social support networks through scheduled reminders to help support pregnant women to utilize maternity services in rural Uganda. The application proposes a type 1 hybrid implementation-effectiveness trial to evaluate and implement the Support-Moms intervention into routine care. Followed by a mixed methods approach grounded on Proctor's implementation outcomes framework and the Consolidated Framework for Implementation Research (CFIR) to evaluate implementation, service and client outcomes, and explore implementation strategies for future scale-up as well as a cost-effectiveness analysis. The proposal has several strengths noted. The revised approach presents a rigorous approach study design, metrics, data collection, and data analysis plan. First, the application addresses an area of high public health significance (maternal mortality and maternal health) with solid premise of effectiveness based on preliminary work. Second, a strong mixed methods approach grounded on implementation outcomes. Third, a sound assessment of costs and cost-effectiveness of implementing Support-Moms into routine care adds an important dimension for potential scaling. No major weakness was noted. Thus, this application has the potential for a major impact.

1. Significance:

Strengths

- Antenatal care (ANC) and skilled births are mainstays of preventing maternal and perinatal morbidity and mortality, particularly in low-income countries (major)
- Ugandan women have low ANC use and skilled births, resulting in one of the highest maternal mortality ratios and perinatal mortality rates in the world. (major)
- Mobile health (mHealth) interventions can support individuals to internalize risks, need, and benefits of health services with high intervention delivery success. (major)
- In prior work (K award), the PI developed a mHealth intervention and the pilot data about its potential effectiveness and acceptability and feasibility is extremely compelling (major)
- There is little data on effectiveness, appropriateness, feasibility, fidelity and incremental costs needed to adopt, or scale up mHealth in sub–Saharan Africa, where the public health impact of such interventions is likely to be the greatest. (major)

Weaknesses

- None noted

2. Investigator(s):

Strengths

- The PI, Dr. Atukunda, has successfully led large maternal health trials before. She has extensive experience with research on mHealth interventions. She has excellent connections to the proposed setting and topic. (major)
- Co-investigators bring experience building implementation and other research capacity in Uganda (Obua, Tita), with maternal health cost-effectiveness research (Waiswa, Nghiem), obstetric practice and implementation in Uganda (Mugyenyi), randomized trials and implementation research (Siedner, Haberer), study design and management (Matthews) and a biostatiscian (Atuhumuza) in Uganda. (major)
- A multi- disciplinary team brings together exceptional expertise and collaboration to conduct the proposed application. Strong team in Uganda with longstanding partnership with the Uganda Ministry of Health, increasing the feasibility and impact of the application. (major)
- A strong collaborative capacity and partnership is well described where many on the team have already worked together and serve/served as mentors to the PI (major)

Weaknesses

- None noted

3. Innovation:

Strengths

- Testing a tailored, sustainable intervention platform to delivery a multi-level intervention designed to create synergistic impact through low- cost technology (major)

Weaknesses

- None of the design, methods, or concepts themselves are innovative (minor)

4. Approach:

Strengths

- (Aim 1) Well described type 1 hybrid effectiveness- implementation trial to test the effectiveness of the Support-Moms intervention (major)
- Primary and secondary effectiveness outcomes well defined and referenced.
- Baseline questionnaire items provides measures from individual to societal level (socio ecological model)
- Approaches for data collection plan and timeline, data analysis plan and sample size calculation are well described. Risk for missing data and participant retention is presented.
- Sex as a biological variable is addressed since all pregnant people regardless of gender orientation/self-identification will be included and there will be no gender exclusion criteria for the social supporters.
- (Aim 2) The evaluation of the implementation using Proctor and CFIR frameworks seems appropriate to achieve the application goal (major)
- Well described mixed methods approach with clear data collection and implementation metrics such as service and client outcomes.
- Data analysis plan for both quant and qual are grounded in the CFIR to explore implementation strategies for scale-up.
- (Aim 3) The cost and cost-effectiveness of implementing Support-Mom's intervention into routine care will be and implications for sustainability will be assessed (major)
- Measure and record the cost of developing and implementing the intervention (program costs), cost to HCPs from increasing demand for/ utilization of services, and costs to users (intervention participants and their social supporters).
- Measures for estimating incremental cost-effectiveness measures are well defined and described.
- A decision tree model to assess the potential impact of economic, clinical or health outcomes of the Support-Moms intervention against routine care will be developed.

Weaknesses

- None noted by Reviewer

5. Environment:

Strengths

- Mbarara University of Science & Technology (MUST) is a local university and serve as the lead organization. It is well suited lead institution with appropriate collaboration to lead this project (major)
- The MUST-HARVARD-MGH Collaboration have an almost 20-Year history of quality research collaboration currently running more than 30 projects worth over US$7million in NIH investment annually and generating (major)

- Junior faculty mentorship and capacity building is outlined, which is innovative and it is a secondary benefit to the context (Moderate).

Weaknesses

- None noted

Study Timeline:

Strengths

- Timeline outlines the activities described in the approach.

Weaknesses

- None

Protections for Human Subjects:

Acceptable Risks and/or Adequate Protections

Data and Safety Monitoring Plan (Applicable for Clinical Trials Only):

Acceptable

Inclusion Plans:

- Sex/Gender: Distribution justified scientifically
- Race/Ethnicity: Distribution justified scientifically
- For NIH-Defined Phase III trials, Plans for valid design and analysis: Not applicable
- Inclusion/Exclusion Based on Age: Distribution justified scientifically

Vertebrate Animals:

Not Applicable (No Vertebrate Animals)

Biohazards:

Not Applicable (No Biohazards)

Resubmission:

- This R01 is a revised resubmission incorporating reviewers comments. The approach has been revised and it presents a rigorous approach study design, metrics, data collection, and data analysis plan. The application addresses an area of high significance (maternal health) with solid premise of effectiveness based on preliminary work. The application includes major changes including the addition of a biostatistician, updated implementation metrics based on Proctor and CFIR-informed implementation strategy, addition of a Data and Safety Monitoring Committee (DSMC) and several improvements in the approach section.

Applications from Foreign Organizations:

Justified

- Compelling rationale: Ugandan women still have low rates of ANC utilization and skilled births, resulting in one of the highest MMR and world perinatal mortality rates in the world

Resource Sharing Plans:

Not Applicable (No Relevant Resources)

Authentication of Key Biological and/or Chemical Resources:

Not Applicable (No Relevant Resources)

Budget and Period of Support:

Recommend as Requested

CRITIQUE 2

Significance: 1
Investigator(s): 2
Innovation: 3
Approach: 1
Environment: 1

Overall Impact: The proposed work addresses the very significant issue of high rate of maternal and perinatal mortality rate in Uganda and overall impact is high because the proposed work is likely to help prevent stillbirths, neonatal death, and maternal deaths. The research is based on very strong prior pilot research by the PI. The project is not very novel, although this is not considered a significant weakness as the use of mHealth in combination with support services in order to increase ANC and maternity services is still not often done. The team has a history of working together in this area. The environment is strong with strong support from the local MOH. The approach has strong aspects in that it combines mHealth with the use of social networks, use of Proctor and CFIR frameworks, and the cost analysis is superb. The resubmission has mostly been responsive to prior critique and strengthened the statistical analysis, increased efforts for several investigators, and added a biostatistician. The DSMP however is still significantly underdeveloped and importantly is missing stopping rules. In conclusion, the potential overall impact of the proposed work is high as it has the potential to increase ANC and maternity services utilization in order to reduce maternal and perinatal mortality and morbidity of Ugandan pregnant individuals.

1. Significance:

Strengths

- The study is poised to address the urgent need to evaluate interventions to increase ANC and maternity service utilization in order to reduce maternal and perinatal mortality and morbidity of Ugandan pregnant individuals.
- Prior research supporting the proposed work includes robust preliminary results from the PI's K43 that suggest that the mHealth intervention was useful, actionable and easy to use and it helped the pregnant participants cope and act within a trusted and friendly environment. Involvement of supporters helped them mobilize support when needed.

- Given the high rate of maternal and perinatal mortality rate in Uganda, if successful the proposed work has the potential to help prevent stillbirths, neonatal death, and maternal deaths.
- The use of mHealth and social supports have proven to be impactful in similar settings.
- Resource requirements and cost of the interventions have been carefully considered.

Weaknesses

- None noted

2. Investigator(s):

Strengths

- The PI is an early-stage investigator with 10 years' experience working in reproductive health in Uganda. She is an epidemiologist with experience in mixed methods research and has led clinical trials in this area.
- The team has an 8-year history of working with each other in this area of research.
- Many of the team's members, including the PI are geographically embedded in Uganda.
- The team is made up of individuals with relevant expertise, including epidemiologists, health economists, maternal-health fetal medicine, and implementation science.
- The resubmission is responsive to the earlier critique and has:
- Added a senior biostatistician
- Increased effort for collaborators Drs. Mugyenyi, Obua, Matthews, Siedner and Nghiem to promote collaboration and direct support of research activities

Weaknesses

- Several biosketches did not conform to the current norm (Waisang, Mugyenyi, Tita, Atuhumuza)

3. Innovation:

Strengths

- This is a multi-level, home grown intervention using low cost technology (moderate)
- The proposed work could speed the translation of research into practice and have a significant positive impact on MMR (major)
- The resubmission is responsive to the earlier critique and has better clarified the innovation of the proposed work (moderate):
- few mHealth interventions have been in the reproductive health field
- fewer mHealth interventions have been evaluated at scale in the public sector.

Weaknesses

- User centric development of mHealth is not considered novel anymore. References cited are from 2015 and 2016; the development of mHealth has fast forwarded since then. Four (systematic) reviews have been published since 2020. (moderate)

4. Approach:

Strengths

- The applicants demonstrate sufficient understanding of dissemination and implementation research principles.
- Thoughtful consideration for contamination
- Appropriate use of Proctor and CFIR frameworks for evaluation of the implementation
- SMS system is tied to local IT vendor and nicely describes how SMS, voice and social network are tied together. The platform is flexible and scalable.
- Clear primary outcome with appropriate complementary secondary outcomes
- The resubmission is responsive to the prior critique:
- The study has been restated as a Hybrid Type I trial
- Timepoints of data and outcomes collection has been corrected
- Reimbursement for transport has been clarified
- Information on financial independence and decision-making autonomy of the participants was added
- Implementation metrics and the CFIR-informed implementation strategy was updated.
- The researchers clarify that that the study will not be powered to demonstrate statistical significance in these secondary analyses.
- Information is provided on how information gained in the qualitative interviews and assessments will be used to develop implementation strategies.
- Sustainability is well defined as an outcome and acceptability will now be measured with the various participants groups
- The statistical analysis plan has been revised to be a single, multivariable logistic regression model. Missing data is addressed.

Weaknesses

- None noted

5. Environment:

Strengths

- Strong support from the Uganda MOH
- Longstanding partnership with the Uganda MOH and the participating sites in Uganda
- Strong evidence of institutional support from MUST, MGH, and UAB, to help sustain the implementation strategies
- All institutions are willing and able to support the proposed work

Weaknesses

- None noted

Study Timeline:

Strengths

- Acceptable for the proposed work; well defined

Weaknesses

- None noted

Protections for Human Subjects:

Acceptable Risks and/or Adequate Protections

Data and Safety Monitoring Plan (Applicable for Clinical Trials Only):

Acceptable

- The now added DSMP is quite sparse. Especially sections are missing describing Data Quality (stopping rules, designation of an independent monitor, subject accrual and compliance, data quality and management) and Safety Review (study reports, study activities review, data handling, data security, etc.).

Inclusion Plans:

- Sex/Gender: Distribution justified scientifically
- Race/Ethnicity: Distribution justified scientifically
- For NIH-Defined Phase III trials, Plans for valid design and analysis: Not applicable
- Inclusion/Exclusion Based on Age: Distribution justified scientifically

Vertebrate Animals:

Not Applicable (No Vertebrate Animals)

Biohazards:

Not Applicable (No Biohazards)

Resubmission:

- The resubmission is responsive to the prior critique.

Applications from Foreign Organizations:

Justified

- The setting of the proposed work in Uganda is justified given the high rate of maternal and perinatal death.

Select Agents:

Not Applicable (No Select Agents)

Resource Sharing Plans:

Acceptable

- Acceptable as submitted

Authentication of Key Biological and/or Chemical Resources:

Not Applicable (No Relevant Resources)

Budget and Period of Support:

Recommend as Requested

CRITIQUE 3

Significance: 1
Investigator(s): 1
Innovation: 1
Approach: 1
Environment: 1

Overall Impact: This proposal seeks to test the effectiveness of a mhealth platform delivering SMS/voice/social support experiences to pregnant women in Uganda to enhance the receipt of ANC and skilled births. The scientific premise is strong with a clear need to close gaps in ANC receipt and promise of mhealth tools in this scenario. In addition, the team has done strong preliminary research to demonstrate the feasibility and preliminary impact of their novel approach. Sex as a biological variable is addressed. The scientific rigor of the proposal is strong with a well-designed RCT with important and relevant primary/secondary outcomes. The revised proposal more clearly maps how Proctor and CFIR frameworks drive the implementation assessment plan. The cost-effectiveness component is an additional strength for a Type I study looking to provide public policy grade evidence to drive change. The revisions have been responsive to the original concerns with regard to the statistical approach, effort allocation for the primary study team and more details around the social supporter roles and evaluation of them. In summary, this is an excellent proposal that generates strong enthusiasm for its innovative approach and is well situated to provide results that could positively impact the health of pregnant moms in Uganda and beyond.

1. Significance:
Strengths

- Clear need to scale up successful interventions for ANC in Uganda in setting of improved capacity of skilled birth providers.
- mHealth and social support (and their combination) have demonstrated impact in many settings and hold promise for ANC but have never been applied for maternal outcomes by a governmental partnership in Uganda
- Very strong preliminary data supporting intervention development and preliminary impact. This proposal really is the next logical step. The pilot testers seem to have really contributed to the development and were satisfied with the product.

Weaknesses

- None

2. Investigator(s):

Strengths

- Very skilled investigator team, led by junior but well-trained PI who is now adequately supported both locally and with international partners enhancing the feasibility for smooth conduct of the study

Weaknesses

- None

3. Innovation:

Strengths

- Novel combination of SMS, audio, social with a very flexible and pragmatic tech stack for dealing with variable tech accessibility and literacy in the community
- Local developer with experience in the community and with the tech limitations along with user centered development have produced an innovative platform ready to be tested

Weaknesses

- None

4. Approach:

Strengths

- Great government partnerships to enhance feasibility
- Clear, important primary outcomes
- Great IT partnership
- Responsive description of SS partners have added clarity to this important component
- Pilot data shows great engagement from SS, a real strength
- Enhanced description of mixed methods is an improvement, now much clearer how imp sci frameworks will be used to guide implementation and evaluation and better description of the qualitative components in terms of methods and value to the project

Weaknesses

- None

5. Environment:

Strengths

- Strong experience and collaboration to support junior PI

Weaknesses

- None

Study Timeline:

Strengths

- Appropriate

Weaknesses

- None

Protections for Human Subjects:
Acceptable Risks and/or Adequate Protections
Data and Safety Monitoring Plan (Applicable for Clinical Trials Only):
Acceptable

Inclusion Plans:
- Sex/Gender: Distribution justified scientifically
- Race/Ethnicity: Distribution justified scientifically
- For NIH-Defined Phase III trials, Plans for valid design and analysis: Not applicable
- Inclusion/Exclusion Based on Age: Distribution justified scientifically

Vertebrate Animals:
Not Applicable (No Vertebrate Animals)

Biohazards:
Not Applicable (No Biohazards)

Resubmission:
- Very responsive application that adequately addressed the prior reviewer concerns for what was already a well scored application. They made adjustments to the statistical plan, the implementation measurement description and the budget allocation to convincingly improve the application and make it well suited to answer its research aims.

Applications from Foreign Organizations:
Justified

Resource Sharing Plans:
Acceptable

Authentication of Key Biological and/or Chemical Resources:
Not Applicable (No Relevant Resources)

Budget and Period of Support:
Recommend as Requested

THE FOLLOWING SECTIONS WERE PREPARED BY THE SCIENTIFIC REVIEW OFFICER TO SUMMARIZE THE OUTCOME OF DISCUSSIONS OF THE REVIEW COMMITTEE, OR REVIEWERS' WRITTEN CRITIQUES, ON THE FOLLOWING ISSUES:

PROTECTION OF HUMAN SUBJECTS: ACCEPTABLE

INCLUSION OF WOMEN PLAN: ACCEPTABLE

INCLUSION OF MINORITIES PLAN: ACCEPTABLE

INCLUSION ACROSS THE LIFESPAN: ACCEPTABLE

COMMITTEE BUDGET RECOMMENDATIONS: The budget was recommended as requested.

Footnotes for 1 R01 HD111692-01A1; PI Name: Atukunda, Esther Cathyln

NIH has modified its policy regarding the receipt of resubmissions (amended applications).See Guide Notice NOT-OD-18-197 at https://grants.nih.gov/grants/guide/notice-files/NOT-OD-18-197.html. The impact/priority score is calculated after discussion of an application by averaging the overall scores (1-9) given by all voting reviewers on the committee and multiplying by 10. The criterion scores are submitted prior to the meeting by the individual reviewers assigned to an application, and are not discussed specifically at the review meeting or calculated into the overall impact score. Some applications also receive a percentile ranking. For details on the review process, see http://grants.nih.gov/grants/peer_review_process.htm#scoring.
